# Supplementary material for: Minimally Invasive Versus Open Distal Gastrectomy for Locally Advanced Gastric Cancer: Trial Sequential Analysis of Randomized Trials
Source: Cancers (Basel). 2024 Dec 6;16(23):4098. doi: 10.3390/cancers16234098 (PMC11640675; doi:10.3390/cancers16234098)
Supplement: Supplementary file 1 [file cancers-16-04098-s001.zip › Suppl. Figure S1.pdf]

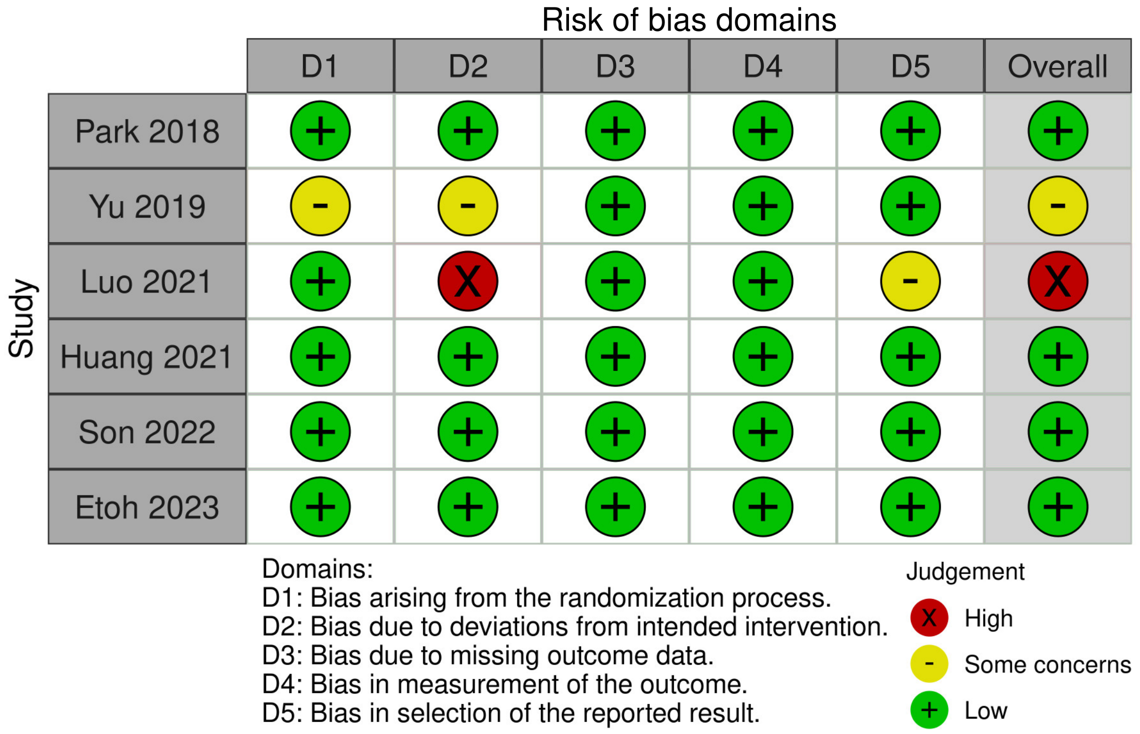

A

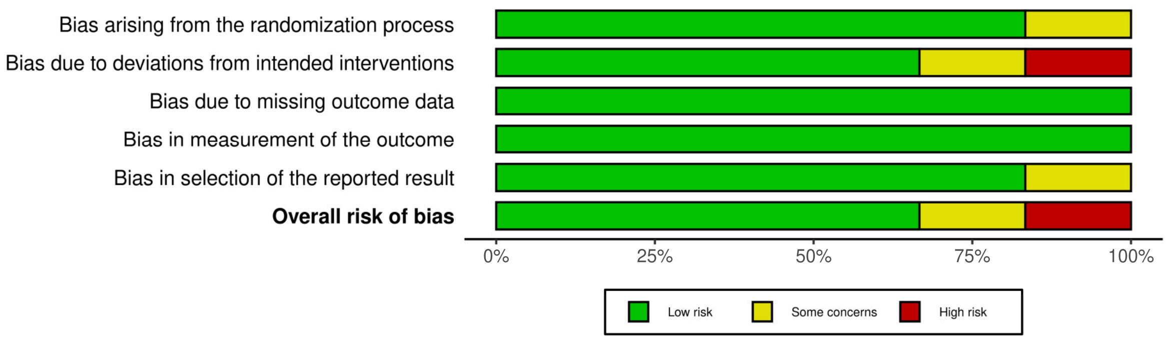

B

Supplementary Figure S1. Cochrane Risk of Bias (ROB2) evaluation. A risk of bias summary; B. risk of bias graph.
