# Supplementary material for: Minimally Invasive Versus Open Distal Gastrectomy for Locally Advanced Gastric Cancer: Trial Sequential Analysis of Randomized Trials
Source: Cancers (Basel). 2024 Dec 6;16(23):4098. doi: 10.3390/cancers16234098 (PMC11640675; doi:10.3390/cancers16234098)
Supplement: Supplementary file 1 [file cancers-16-04098-s001.zip › Suppl. Table S1.pdf]

Author, year country	Study design	Randomization method	Surgeons' eligibility	Surgical quality control	ITT/PP	Power analysis
Park et al., 2018, Korea	nr	Web-based randomization system with fixed block size 1:1 randomization	>30 LADG	Surgery recorded for evaluation	ITT	Y(UP)
Yu et al., 2019, China	non-inferiority RCT	For randomization, a central dynamic, stratified strategy was adopted. The randomization sequence was generated using the Pocock-Simon minimization method	15 experienced surgeons	nr	ITT/PP	Y (DFS)
Luo et al., 2021, China	nr	Envelope method	nr	nr	nr	nr
Huang et al., 2021, China	non-inferiority RCT	For randomization, a central dynamic, stratified strategy was adopted. The randomization sequence was generated using the Pocock-Simon minimization method	Experienced surgeon in high center volume	nr	ITT/PP	Y (DFS/OS)
Son et al., 2022, Korea	non-inferiority RCT	Patients were randomly assigned (1:1) to undergo laparoscopic or open surgery	Video assessment form – surgeons having performed >50 MIDG & >50 ODG	Independent quality control study was performed to qualify the participating surgeons (KLASS-02-QC)	ITT	Y (DFS)
Etoh et al., 2023 Japan	non-inferiority RCT	Group assignment was conducted using the minimization method according to the clinical depth of invasion (MP vs SS/SE), clinical N category (N0 vs N1 vs N2), and the institution	Experienced surgeons (ESSQS certification)	Photographs operative field and lymphadenectomy	ITT/PP	Y (DFS)

Supplementary Table S1. Randomized Clinical Trials (RCTs) quality evaluation. Open Distal Gastrectomy (ODG) and minimally invasive distal gastrectomy (MIDG). P patient, S surgeon, Stat statistician, Y yes, UP under-powered, JSES Japan Society for Endoscopic Surgery.
