# Supplementary material for: Minimally Invasive Versus Open Distal Gastrectomy for Locally Advanced Gastric Cancer: Trial Sequential Analysis of Randomized Trials
Source: Cancers (Basel). 2024 Dec 6;16(23):4098. doi: 10.3390/cancers16234098 (PMC11640675; doi:10.3390/cancers16234098)
Supplement: Supplementary file 1 [file cancers-16-04098-s001.zip › Suppl. Table S2.pdf]

Certainty assessment							№ of patients		Effect	Certainty	Importance
**№ of studies**	**Study design**	**Risk of bias**	**Inconsistency**	**Indirectness**	**Imprecision**	**Other considerations**	**[intervention]**	**[comparison]**	**Relative (95% CI)**		
**5-year OS**											
4	randomised trials	not serious	not serious	not serious	not serious	all plausible residual confounding would reduce the demonstrated effect	1318	1327	**HR 0.86** (0.70 to 1.04)	⨁⨁⨁⨁ High	CRITICAL
**5-year DFS**											
5	randomised trials	not serious	not serious	not serious	not serious	all plausible residual confounding would reduce the demonstrated effect	1414	1421	**HR 1.03** (0.87 to 1.38)	⨁⨁⨁⨁ High	CRITICAL

Supplementary Table S2. CI: confidence interval; **HR:** hazard ratio
